# Case of Inherited Partial AZFa Deletion without Impact on Male Fertility

**DOI:** 10.1155/2019/3802613

**Published:** 2019-10-31

**Authors:** Baiba Alksere, Dace Berzina, Alesja Dudorova, Una Conka, Santa Andersone, Evija Pimane, Sandra Krasucka, Arita Blumberga, Aigars Dzalbs, Ieva Grinfelde, Natalija Vedmedovska, Violeta Fodina, Juris Erenpreiss

**Affiliations:** ^1^Genetic Laboratory, Clinic “IVF-Riga”, Riga, Latvia; ^2^Department of Gynecology and Reproduction, Clinic “IVF-Riga”, Riga, Latvia; ^3^Center of Medical Genetics and Prenatal Diagnostics, Children's Clinical University Hospital, Riga, Latvia; ^4^Riga Stradins University, Latvia; ^5^Department of Andrology, Clinic “IVF-Riga”, Riga, Latvia

## Abstract

Male factor infertility accounts for 40–50% of all infertility cases. Deletions of one or more AZF region parts in chromosome Y are one of the most common genetic causes of male infertility. Usually full or partial AZF deletions, including genes involved in spermatogenesis, are associated with spermatogenic failure. Here we report a case of a Caucasian man with partial AZFa region deletion from a couple with secondary infertility. Partial AZFa deletion, involving part of *USP9Y* gene appears to be benign, as we proved transmission from father to son. According to our results, it is recommended to revise guidelines on markers selected for testing of AZFa region deletion, to be more selective against *DDX3Y* gene and exclude probably benign microdeletions involving only *USP9Y* gene.

## 1. Background

Male factor infertility accounts for 40–50% of all infertility cases. Deletions of one or more AZF region parts in chromosome Y are one of the most common genetic causes of male infertility. Microdeletions of AZF regions in Y chromosome occur as de novo event in 2–10% nonobstructive azoospermia or oligospermia cases [1–3]. Usually full or partial AZF deletions, including genes involved in spermatogenesis, are associated with spermatogenic failure. Entire AZFa deletions are induced by recombination between HERV15 class proviruses with ~800 kb distance [4–6]. This sort of Y chromosome's microdeletions might be the cause of Sertoli cell only (SCO) syndrome – a total lack of spermatogenesis [7]. There are several events reported about the inheritance of different AZF regions' deletions from fathers to their sons [8–10]. Real time polymerase chain reaction, using STS markers as stated by the European Academy of Andrology (EAA) and the European Molecular Genetics Quality Network (EMQN), is a convenient screening method to detect these changes [11]. Unfortunately, this testing approach might give false positive results due to single nucleotide polymorphisms (SNPs) in primer annealing site (peculiar for Asian populations) [12]. Also, conventional screening for AZFa and AZFb deletions includes PCR of two STS markers (sY84 and sY86 for AZFa region, sY127 and sY134 for AZFb region), which do not flank genes, important in sperm development, from both sides. Therefore, false positive outcome of AZFa or AZFb deletion might appear, as the test detects only the absence of these STS markers, not the absence of genes.

Here we report a case of a Caucasian man with partial AZFa region deletion from a couple with secondary infertility. Aim of the study was to evaluate the size and involved genes of partial AZFa region deletion detected in a man with normal semen analysis.

## 2. Case Presentation

Patient at the time of appointment in fertility clinic was 31 years old, healthy individual. His partner was 27 years old woman with one missed pregnancy at week 6 in anamnesis. All tested hormonal and biochemical markers – prolactin, vitamin D, testosterone, T4 and TSH — were in normal range. Semen analysis did not show significant changes in volume, concentration and sperm motility. The count of the round cells—1mil/ml, did not exceed reference levels of normal criteria. Percentage of morphologically normal spermatozoa was slightly deteriorated — 4% cells with normal morphology, amorphous sperm heads, altered acrosomal distribution and enlarged necks. Sperm DNA fragmentation test did not show increased sperm DNA damage, fragmented DNA occurred in 13% of cells by HaloKit test (<16% DNA fragmentation is associated with normal fertility level). The patient was referred for AZF microdeletion testing after karyotype analysis, which revealed normal 46, XY karyotype, but showed slightly decreased levels of constitutive heterochromatin in the long arm of chromosome Y.

Genomic DNA was extracted, using Magpurix Blood DNA Extraction Kit 200, according to the manufacturer's protocol. Analysis of AZF region deletions was performed using AZF System Y-chromosome (Sacace) reagents' kit. This Real Time PCR test is based on the amplification of two, specific, nonpolymorphic STS locuses in each region of Y chromosome – AZFa, AZFb and AZFc. The STS locuses used in this kit for detection of AZF regions deletions are: AZFa: sY84, sY86, AZFb: sY127, sY134, AZFc: sY254, sY255, as recommended in the guidelines of American and European Andrology societies (ref…). The sensitivity of this method is >95%.

Analyzing patient's DNA, amplification of STS markers AZFa: sY86, AZFb: sY127, sY134, AZFc: sY254, sY255, were detected, so these regions are all present in patient's genome. Amplification product of AZFa: sY84 was absent, and deletion of AZFa regions' STS sY84 was confirmed by repeated analysis.

As full deletion of AZFa region is linked with severely impaired spermatogenesis, but patient had normal semen analysis with only slight deviations, more detailed analysis of AZFa region was performed.

To exclude SNP in region of primer binding sites of sY84, PCR with redesigned primers and subsequent capillary electrophoresis were performed. Primers were as described previously, PCR conditions are available under request (Table 1, [12]). Normal control DNA from male without AZFa deletion, had amplification product and sequence of AZFa region was obtained, but patients' DNA did not amplify PCR product and sequencing was not possible. Therefore, it was concluded, that patient has deletion in AZFa region.

For determining the size of deletion, markers and PCR primers, specific for distinct locations in AZFa region, were found, using MSY Breakpoint Mapper (Table 1, [13]). This database of sequence-tagged sites (STSs) gives a possibility for more precise mapping of deletions in the male-specific region of the human Y chromosome.

PCR assays for all markers to determine the approximate size of deletion and involved genes (Table 1) were performed, using nuclease free water, 10x PCR buffer (20 mM MgCl2), 10 mM dNTP, betaine, 10 nM primer mixes and 1U/*µ*l Taq polymerase. PCR conditions are available under request.

Analysis of patients' father DNA was performed to investigate, whether the AZFa deletion is passed from father to son.

**Figure 1:**
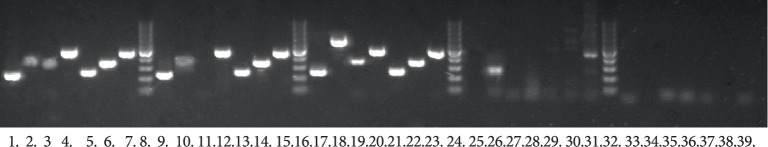
PCR products of all tested AZFa region markers. 1000 bp ladder was used as the size control (lanes 8, 16, 24, 32). Lanes 1–7: Patient's DNA sample. Lanes 9–15: DNA sample of the father of patient. Lanes 17 – 23: Positive control (healthy male without AZF deletions). Lanes 25–31: Negative control (male with full AZFa deletion). Lanes 33–39: No template control. For each sample, PCR products representing the tested markers are shown in following order: sY85: lanes no. 1, 9, 17, 26, 33; sy84: lanes no. 2, 10, 18, 26, 34; sY1323: lanes no. 3, 11, 19, 27, 35; USP9Y-44: lanes no. 4, 12, 20, 28, 36; sY87: lanes no. 5, 13, 21, 29, 37; sY1234: lanes no. 6, 14, 22, 30, 39; DDX3Y-16: lanes no. 7, 15, 23, 31, 39.

Deletion of both Y chromosomes' STSs sY84 and sY1323 was detected in patients' (Figure 1, lanes 1–7) and his father DNA samples (lanes 9–16), when compared to positive control (healthy male DNA) (lanes 18–24), confirming the inheritance of this region's deletion. None of markers were visualized from DNA of infertile male with full AZFa region deletion (lanes 26–32).

**Figure 2:**
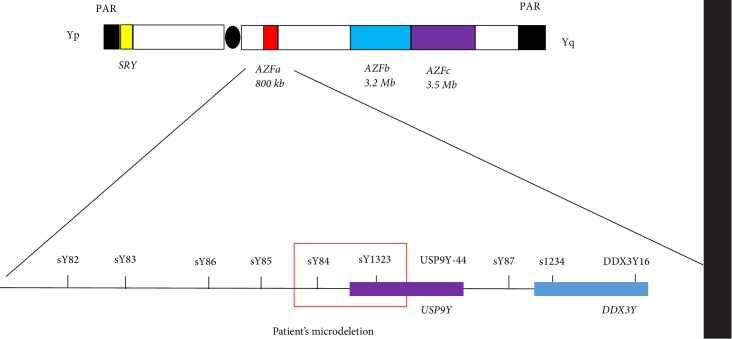
Schematic drawing of the tested STS markers. Partial deletion of AZFa is marked with red box.

The deletion spans sY84 and sY1323 markers, removing part of one of the AZFa region gene *USP9Y* and leaving second AZFa gene *DDX3Y* intact (Figure 2). AZFa partial microdeletion was detected, removing part of AZFa non-coding region and 5ʹ part of *USP9Y* gene, as testing *USP9Y* deletion with STS markers in both ends of the gene, sY1323 and *USP9Y*-44, showed, that 3ʹ marker USP9Y-44 was present. Moreover, STS sY84 — a marker upstream the *USP9Y*, and sY1323 — a marker in 5ʹ start of the gene, were absent, and this result confirms partial AZFa and *USP9Y* deletion. Exact breakpoints were not mapped, and sequencing of full AZFa region was not performed due to technical issues. Also, the deletion was already proven with marker testing, and it was not necessary to do full AZFa region investigation. As results of marker testing was the same for father of patient, we proved germline transmission of deletion from father to son and excluded *de novo *event.

## 3. Discussion

In this case report, germline transmitted Y chromosomes' AZFa partial microdeletion was detected, removing part of AZFa noncoding region and 5ʹ part of *USP9Y* gene. Partial AZFa deletions not always affects spermatogenesis, and sperm production in these cases might be essentially impaired only, if aberrations of one AZFa gene *DDX3Y*, are involved. In this case, the deletion did not affect sperm cell production, as almost all the sperm quality parameters were in normal range. There are evidences, that *USP9Y* has a role in spermatogenesis, changes in it is related to mild oligozoospermia phenotypes and are compatible with fertility [14, 15]. Liu et al. in a comprehensive research found partial AZFa microdeletion, including loss of marker Y86, from nt 14469266 to 14607672 in sub-fertile father and son [16]. Luddi et al. has demonstrated that one of the two AZFa region genes, *USP9Y*, deletion is not a major cause of oligoastenozoospermia or azoospermia, and carriers of this microdeletion have normal sperm parameters [17]. Also, missense variants of the *USP9Y* do not have constitutive impact on male infertility. Banks et al. performed in silico analysis of *USP9Y* variants and found out that all males with predicted protein damaging variants were able to have siblings without assisted reproductive therapies, therefore establishing the fact, that *USP9Y* has a minor role in gametogenesis [18].

The role of *USP9Y* remains questionable, as in some animals, for example, chimpanzees, this gene expression has stopped, and still their spermatogenesis progresses normally [19]. *USP9Y* has a homolog USP9X with 97% approximate identity and cognate expression in early stages of the development of gametes [20]. Krausz et al. had presented two cases, that AZFa deletions, including *USP9Y*, might be inherited from fertile men to their offsprings. The fertility of these patients was impaired, and moderate oligoasthenoteratozoospermia was observed in both cases, so probably this gene has a fine-tuning role in humans [21]. *USP9Y* might play a role in the post-meiotic stage of human sperm cell growth. In a case of one patient with spermatid arrest, a “de novo” deletion of 4 base pairs, encompassing splice donor site in exon 8, was found [22].

Similarly, to only *USP9Y* aberrations, phenotypes with full AZFa deletions, incorporating *DDX3Y*, show only changes in testicular development, without somatic phenotype alterations [23]. In male germ cells *DDX3Y*, which is usually omnipresent, undergoes strict control of translation in testis, and this specific transcript is mainly related to differentiation of spermatogonia and spermatocytes – premeiotic germ cells. According to this, *DDX3Y* should be regarded as the main AZFa region gene participating in sperm cell development [24].

The marginal role of *USP9Y* in sperm production may be explained by the replacing mechanisms of this gene homologs on X chromosome or autosomes, or other compensatory processes [14]. In contrast to *USP9Y*, *DDX3Y* function cannot be dispensable by its homologue, DDX3X. Although DDX3X expression occurs globally in mice testis, they might be differentially regulated [25].

## 4. Conclusion

Our results confirm the fact, that partial deletion of AZFa region and *USP9Y* does not affect fertility, as we have proved inheritance of the deletion from father to son, in father there are no documented fertility problems. We conclude that partial deletions of AZFa region should be studied in details, to exclude or confirm involvement in impaired spermatogenesis, as only full deletion of AZFa region could be marker for infertility. It is highly advisable to choose markers of AZFa region, which can confirm deletion of genes related to spermatogenesis. According to our results, it is recommended to revise guidelines on markers selected for testing of AZFa region deletion, to be more selective against *DDX3Y* gene and exclude probably benign microdeletions involving only *USP9Y* gene.

## Figures and Tables

**Table 1:** Markers, used for the detection of AZFa region deletion size (#Wu et al. [12], ^∗^MSY Breakpoint Mapper [13]), F — forward, R — reverse.

STS	Genomic coordinates (GRCh38)	Primer	Size of expected PCR product
sy85^∗^	Y:12525881–12526250	F — TGGCAATTTGCCTATGAAGT	369 bp
		R — ACAGGCTATTTGACTGGCAG	
sY84#	Y:12678105–12678432	F — GCTGAGGAGTTGTGGAGACC	642 bp
		R — GCAAGGACATTCCAGGGTTA	
sY1323^∗^ (intragenic)	Y:12721978–12722325	F — ATGGTGAATATAATATAGGCAGAATTT	348 bp
		R — CCTTACCAGGAAGGTTTGTGA	
*USP9Y*-44^∗^ (intragenic)	Y:12856484–12856970	F — CCAGATCTTACAGGTGAGGGTTT	487 bp
		R — GCAAACAAAACTGCACATGATT	
sY87^∗^	Y:12897218–12897468	F – TCTGTTGCTTGAAAAGAGGG	251 bp
		R — ACTGCAGGAAGAATCAGCTG	
sY1234^∗^ (intragenic)	Y:12914758–12915107	F — TTACCCCTTTCACCCACTGA	350 bp
		R – CCATAAACTACACAAGGACGAACT	
*DDX3Y*-16^∗^ (intragenic)	Y:12917260-12917716	F — TGGGACATTAATGGGATGGT	457 bp
		R – GTTGCCACCCACCTGTAATC	
